# Supplementary material for: Pattern of New Gene Origination in a Special Fish Lineage, the Flatfishes
Source: Genes (Basel). 2021 Nov 19;12(11):1819. doi: 10.3390/genes12111819 (PMC8618825; doi:10.3390/genes12111819)
Supplement: Supplementary file 1 [file genes-12-01819-s001.zip › genes-1455005-supplementary/Figure S2.pdf]

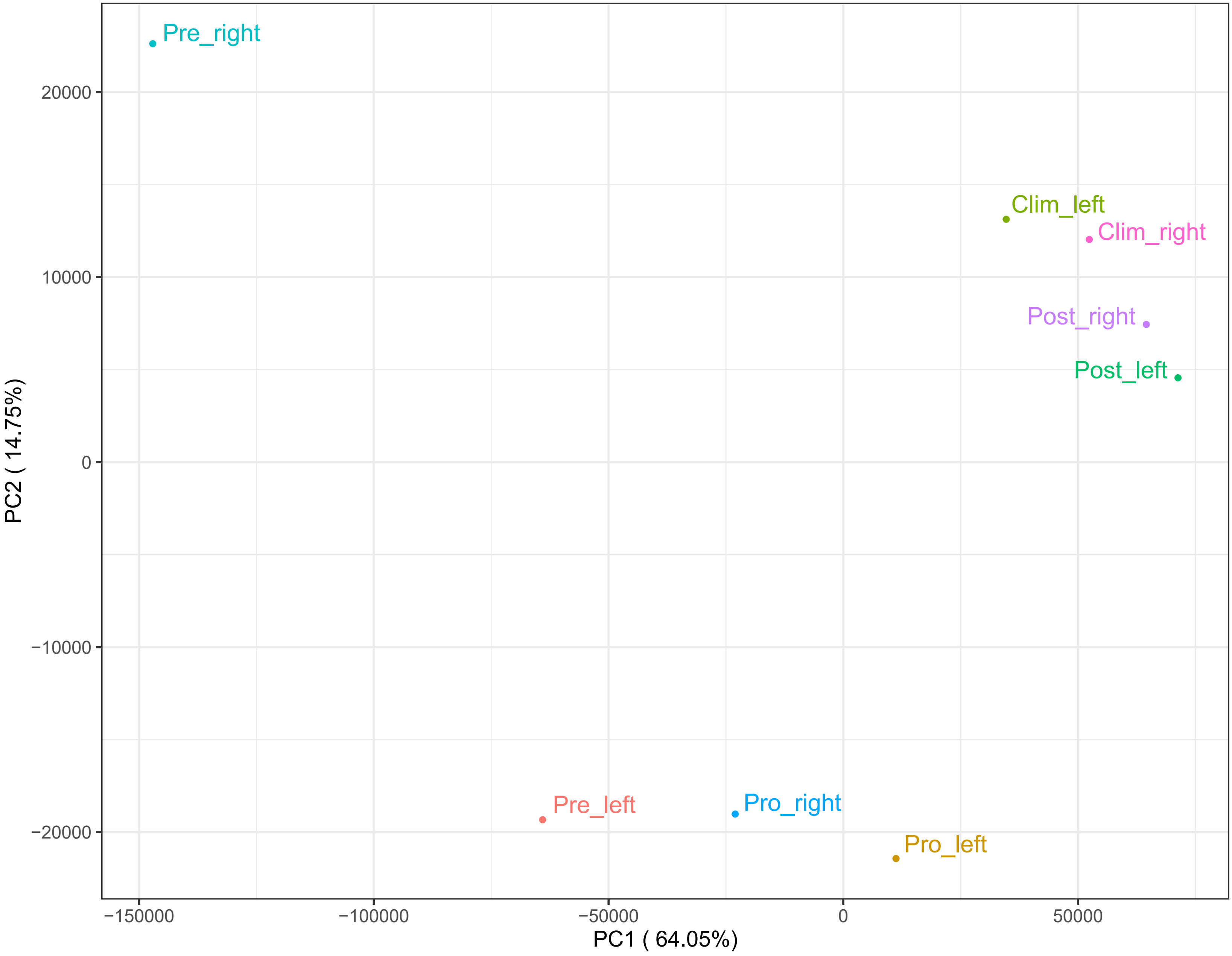


**Figure S2.** The Principal Component Analysis (PCA) plot of muscle expression profiles of different samples of the Japanese flounder, including left and right side at different development stages. Pre, pre-metamorphic larva; Pro, pro-metamorphic larva; Clim, metamorphic climax larva; Post, post-metamorphic larva.
