# Supplementary material for: Pattern of New Gene Origination in a Special Fish Lineage, the Flatfishes
Source: Genes (Basel). 2021 Nov 19;12(11):1819. doi: 10.3390/genes12111819 (PMC8618825; doi:10.3390/genes12111819)
Supplement: Supplementary file 1 [file genes-12-01819-s001.zip › genes-1455005-supplementary/Table S1.pdf]

Table S1. New genes with significant differential expression between the left and right sides.

High expression side	Gene ID	Gene name	*P*-value	$Log_2FoldChange$
Right	evm.model.Hic_chr_16.3	*Nlrc3-like*	5.23e-06	-28.57
Right	evm.model.Hic_chr_17.469	*Ugt1a2*	7.82e-05	-4.84
Right	evm.model.Hic_chr_10.487	*Smarcad1*	1.57e-03	-4.34
Right	evm.model.Hic_chr_18.213	*Hipk1*	2.17e-03	-4.06
Right	evm.model.Hic_chr_14.418	*Ttc21b*	5.14e-03	-3.78
Right	evm.model.Hic_chr_15.251	*Trim25*	5.47e-03	-1.14
Left	evm.model.Hic_chr_8.550	*S100a5-like*	1.54e-05	3.89
Left	evm.model.Hic_chr_6.523	-	4.27e-05	3.42
Left	evm.model.Hic_chr_11.402	*Fel*	1.27e-04	2.41
