# Supplementary material for: Pattern of New Gene Origination in a Special Fish Lineage, the Flatfishes
Source: Genes (Basel). 2021 Nov 19;12(11):1819. doi: 10.3390/genes12111819 (PMC8618825; doi:10.3390/genes12111819)
Supplement: Supplementary file 1 [file genes-12-01819-s001.zip › genes-1455005-supplementary/Table S2.pdf]

Table S2. The branch and number of genes in starry flounder.

Branch	Gene number
-1	3750
0	16803
1	48
2	6
3	47
4	147
5	1341
