# Supplementary figures and images for: Pattern of New Gene Origination in a Special Fish Lineage, the Flatfishes
Source: Genes (Basel). 2021 Nov 19;12(11):1819. doi: 10.3390/genes12111819 (PMC8618825; doi:10.3390/genes12111819)

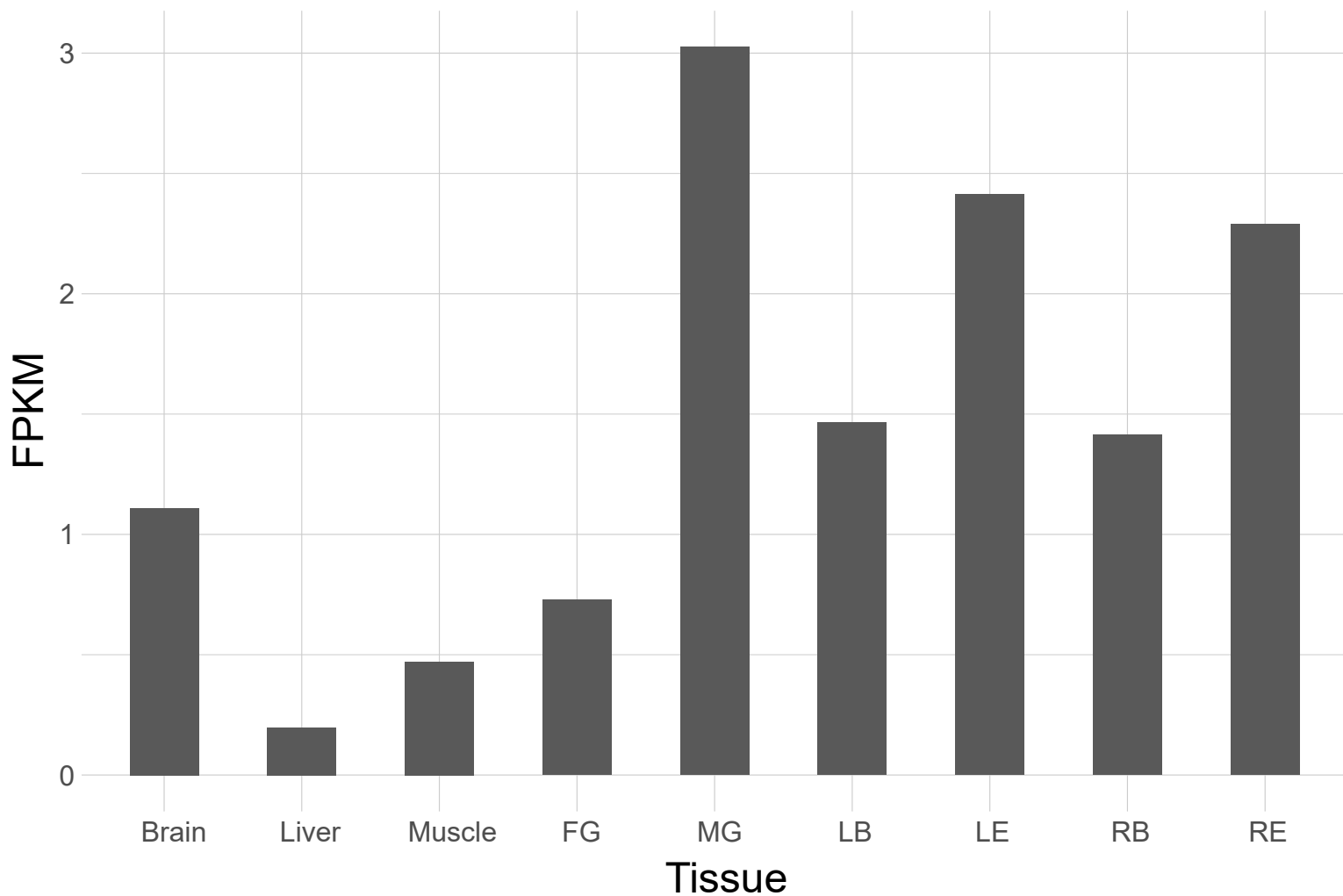

**Figure S1.** Expression profiles of gene Hic\_chr\_10.1034 in different tissues.

Supplement: Supplementary file 1 [file genes-12-01819-s001.zip › genes-1455005-supplementary/Figure S1.pdf]
